# “I was very sad, but not depressed”: phenomenological differences between adjustment disorder and a major depressive episode

**DOI:** 10.3389/fpsyt.2023.1291659

**Published:** 2023-12-11

**Authors:** Juan Pablo Zapata-Ospina, Mercedes Jiménez-Benítez, Marco Fierro

**Affiliations:** ^1^Institute of Medical Research, School of Medicine, Universidad de Antioquia, Academic Group of Clinical Epidemiology (GRAEPIC), Medellín, Colombia; ^2^Hospital Alma Máter de Antioquia, Medellín (Antioquia), Medellín, Colombia; ^3^Department of Psychology, Faculty of Social and Human Sciences, University of Antioquia, Medellín, Colombia; ^4^Department of Psychiatry, School of Medicine, Fundación Universitaria Sanitas, Psychopathology and Society Research Group, Bogotá, Colombia

**Keywords:** adjustment disorders, emotional adjustment, depression, psychopathology, phenomenological study

## Abstract

**Introduction:**

Adjustment disorder (AD) is a diagnosis that must be differentiated from major depressive episode (MDE) because of the therapeutic implications. The aim of this study is to understand the experience of patients who in their lifetime have been diagnosed with AD as well as MDE to establish the characteristics of each disorder.

**Methods:**

A descriptive phenomenological approach was used with in-depth interviews to four patients and the method proposed by Colaizzi to understand the experiences and reach the description of both disorders.

**Results:**

Three women and one man, with advanced schooling were interviewed. The participants emphasized the existence of differences that were grouped in: the attribution made by the individual, the theme of cognitions, the variability in the course, the possibility of mood modulation, the syndrome severity, the presence of hopelessness and the perceived course.

**Conclusion:**

Phenomenological differences were found in the subjective experience of MDE and AD. The MDE would be described as an intense state of generalized shutdown of the subject’s own life, with little response to events, and the AD, as a dynamic reaction attributed to a stressful event, with high variability in the course of symptoms due to the dependence on such event, with the preserved hope that it will end.

## Introduction

Adjustment disorder (AD) has been defined as an individual’s emotional or behavioral reaction to a stressful event. Such reaction is a maladaptive response because it undermines the person’s normal functioning (1). It is one of the most common mental disorders in clinical practice, particularly in high complexity hospitals, where its prevalence reaches up to 18.5%. In addition, AD patients are 12 times as likely to commit suicide as the general population (2) and 5 to 36% of those who have committed suicide had been diagnosed with AD during psychological autopsy (3–7). AD also results in higher usage of healthcare services, higher costs (8) and workplace absenteeism (9).

The mental disorder classification systems establish AD diagnosis on the basis of affective and behavioral symptoms appearing after a stressful event, provided those symptoms do not match the criteria for other disorders such as depression or post-traumatic stress (10). It would thus appear that the defining feature of AD is the relationship with an identifiable stressor, but most psychiatric disorders also exhibit this. Therefore, the essence of AD would be the failure to match the diagnosis criteria for the other disorders, which has led to criticism of its theoretical foundations. A number of authors consider it a disorder with no underlying theoretical model, and a hardly useful diagnosis of exclusion (11–13).

However, AD is the seventh diagnostic category most used by psychiatrists around the world (14) and the eighth for psychologists (15). The experience derived from seeing patients along with the empirical data show that AD is truly an important category (16–20). However, it is necessary to clarify its symptoms and provide better foundations for its diagnosis criteria and the model supporting it. Some authors consider a major depressive episode (MDE) as the differential diagnosis for AD and claim that the difference between these requires further research because of the therapeutic implications (21) and MDE may be clinically indistinguishable from AD when the stressor is still present and the diagnosis can only be confirmed after it disappears along with the symptoms.

Research on some differentiating features has been conducted and the results report that, after a stressful event, AD patients commit suicide earlier than those with MDE (22) in relation to the occurrence of the stressful event. Likewise, they usually are less likely to have a perfectionist personality or a personal/family history of mental disorders (23, 24). They are usually less likely to report maintenance insomnia (25) and may have a different type of emotional processing, as suggested by the differences in the interhemispheric coherence observed in the parietal-occipital area in their electroencephalogram (26). Nevertheless, these are quantitative differences based on the sample distribution of the variable. Thus, they could be of little utility during clinical practice since they can be present to a lesser or greater extend in both AD and MDE patients.

Based on their interaction with patients, some clinicians have reported the existence of qualitative differences. For instance, even though a sad mood can be observed in both disorders, it is persistently poorly modulated in those with MDE. In contrast, sadness in AD depends on the cognitive presence of the stressor, which is why affective reactivity is higher (11). As a result, the experience of sadness may vary for each disorder and there may be essential features that are different from the quantitative approach.

In this scenario, returning to the patients’ narrative of their experiences can be useful as a potential source of refinement of the diagnostic criteria, since the nuances of the daily experience of the symptoms can be found, which will broaden the conceptualization of the clinical manifestations of the different mental disorders. In other words, to return to phenomenology in psychopathology to balance operationalism, with a method of capturing information, such as the in-depth interview, for the understanding we make of the patients’ experience and improve the co-production of clinical care between the patient and the clinician (27–29). This has been the call of many authors in psychiatry (30, 31).

During the clinical practice of the research group, the principal investigator learned of a patient who had first been diagnosed with MDE and later with AD. From that moment onward, the search for patients with similar situations began. The goal was to abstract two experiences that were different at least temporarily and led to different diagnoses. The objective of this study was thus to understand the experiences of those patients who had been diagnosed with AD and MDE throughout their lives to establish the features of the subjective experience with each disorder.

## Materials and methods

We used a phenomenological descriptive approach (32) to understand the features of the experience during the AD and MDE. This approach is grounded on the philosophy of Edmund Husserl, who established that returning to the things themselves, to the structure underlying the experience, extracted from the subjective experience, to the things as they appear to consciousness (33). Phenomenology deals with the ‘eidetic’ underpinnings -or the essence- of how things appear for us (34). From this point of view, the goal is to find the antepredicative elements which contribute to the construction of the meaning of things, actions and the world (35). In our case, the goal should be returning to the experiences of the patients, since this is the raw material with which psychiatrists diagnose patients in their daily practice. Employing imaginative variation and accepting participants’ descriptions of their experiences we can understand the core nature of an experience (36). For this report, we followed the COnsolidated criteria for REporting Qualitative research (COREQ) (37) (Supplementary Table).

### Participants

Purposive sampling was carried out with the help of the psychiatrists and psychologists from our research group in their private or institutional practice. They were contacted by telephone by the principal investigator for the invitation to participate, after a previous introduction by the treating clinician. Regarding selection criteria, we chose Colombian patients with at least two episodes of a condition whose diagnostic label was AD or MDE. Upon finding a candidate, the lead researcher would contact the patient to invite them to participate. In no case did the principal investigator -a psychiatrist himself- provide any form of healthcare to the patients. We pre-specified in the research project that the inclusion of at least four participants, which is within the range of phenomenology and life history studies and even appears as a suggested guideline for PhD projects (38, 39). This sample size was determined, on the one hand, by the limited access to cases having had both diagnoses and, on the other, by the type of in-depth interview with which we aimed to understand the experience and the facility to present -later- the voices of the participants (40). Ultimately, we included the minimum of four participants, as the depth of understanding derived from the participants’ detailed narrative was deemed satisfactory to declare theoretical saturation. We had no refusals to participate.

### Data collection and processing

In-depth interviews were conducted with the participants via videocalls which were later recorded. The participants were at home, without any companion at the time of the interview, as their privacy was pursued. To co-create the experiences, we used the life history methodology (41) to arrange the data chronologically and guide conversation. We hand drew a line representing time in years and sketched the data provided by the participant. After a basic identification, life events were drawn in chronological order, including the presence of mental symptoms, their evolution over time the professional care received. On this basis, we then delved into the perception of symptoms and diagnoses. We aimed to see how the symptoms appeared, the presence of stressful events and the manner in which patients themselves perceived the difference between the experiences giving rise to the diagnosis of AD and MDE. This line was continuously shown to the participant to check the accuracy of our representations (42). Notes of interviewer’s impressions were also made. The interviews were carried out in at least two sessions (with a maximum of five sessions) with each participant and lasted more than 1 h each by the principal investigator (JPZO), a male psychiatrist with a master’s degree in Clinical Epidemiology and a PhD(c) in Clinical Medicine who also has been trained in qualitative research. He was introduced as an external researcher (i.e., not involved in their treatment) and explained that he wanted to learn from the experiences of participants with affective symptoms for his thesis. Each video interview was recorded and transcribed using a code to protect the identity of the participants. The interviewer himself transcribed the interview line by line to become more familiar with the data. The participants themselves did not know anything about our hypothesis stating that there could be a difference between AD and MDE. As a form of *bracketing*, which in Husserl originally implied to see how things appear to us without preconception and reduce or lead back (43), we opted for strategies in data collection (44), with the free narration without presuppositions and without opinions heard from clinicians. Only upon concluding the ultimate interview, we sought their perspective on the regarding that both diagnoses correspond to the same state and so we went back to when we were not convinced of identifying distinctions between the two disorders and were writing the proposal, making a concerted effort to suspend our feeling-intentions.

### Data analysis

For the ipsative assessment, in which individuals compare their own states during MDE and AD, we used the method proposed by Colaizzi (45) in order to understand their situation and obtain a description of the subjective experience of each disorder. After becoming familiar with the interviews, we synthesized the narrative of each individual. Then, we identified the relevant statements about the experiences that led to the diagnoses and assigned them meanings. The principal investigator and a psychologist with a master’s degree and a PhD in clinical psychology (MJB) formulated the meanings of the patients’ statements independently. No major discrepancies were found between the two. Finally, we grouped meanings by topic and built a comprehensive description of the differences between the two experiences to define the features of each disorder. The extent of data saturation was assessed through a concurrent process conducted alongside the data collection. Subsequently, the determination of saturation was established through mutual agreement between both researchers. Theoretical saturation was understood as range (frequency and variety of statements) and complexity of topics (level of synthesis and abstraction of meanings) and the satisfaction of the researchers to have found differences between the two disorders studied sufficiently explanatory (46, 47). To increase credibility, the analysis was supervised by two psychopathology experts (MF one of them) who reviewed the participants’ declarations, the researchers’ interpretations, and the topic grouping. Also, transcripts, analyses and the final draft were disclosed to the participants to verify that we captured their experiences correctly and that they agreed with the details to be published; no corrections were needed. We do not employ software due to preference for manual analysis.

### Ethical considerations

This study complies with the Helsinki Declaration and was approved by the Ethical Review Board of the School of Medicine of the University of Antioquia. In this paper, the identity and personal data of the participants was protected. The contents of the paper were approved by the participants themselves.

## Results

During 2020 we included four participants who were diagnosed with AD and MDE during psychiatry or psychology consultations.

### Sample

#### Participant 1 (P1)

**Figure 1:**
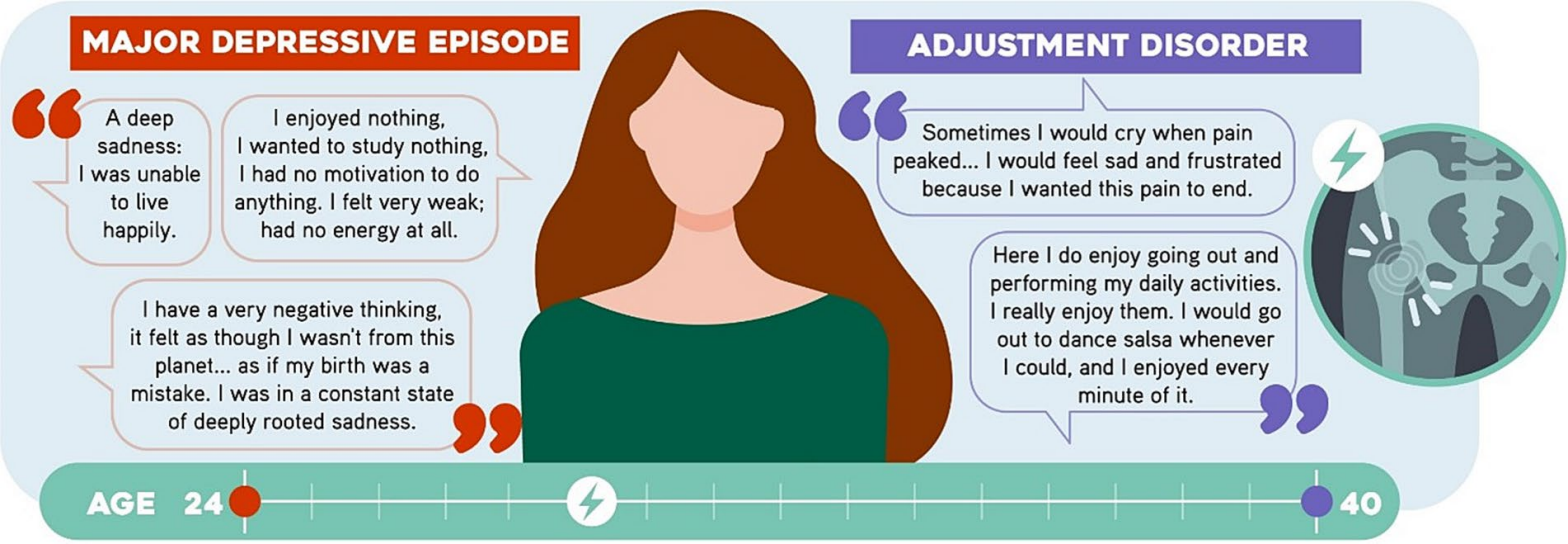
Statements by Participant 1 (P1). Some sentences said by the participant are shown.

A 41-year-old single woman who is a physicist, has no children and is currently studying a master’s degree. At the age of 24, she started to feel sad and cry frequently “for no reason.” She also experienced anhedonia, hypobulia, decreased vitality and sexual desire, ideas of hopelessness and worthlessness, hyporexia and subjective weight loss. As a result, her academic performance decreased. She was taken to a psychiatric outpatient clinic, where she was diagnosed with *major depressive episode* and started treatment with escitalopram. She had sustained clinical recovery and abandoned follow-up and treatment by psychiatry. At the age of 28, she suffered a sports injury to the right inguinal region which resulted in chronic pelvic pain. She described experiencing sadness, irritability, anxiety, low energy, conciliation insomnia and hyporexia. These symptoms caused suffering and impacted her performance at work and in her studies. They also persisted in time and their presentation oscillated based on pain intensity. She was followed-up by physical medicine and rehabilitation and her analgesic treatment consisted of opioids. At the age of 40 she booked an appointment with the same psychiatrist who saw her for the first time. This time the professional considered a diagnosis of *adjustment disorder*. Figure 1 shows some of her statements.

#### Participant 2 (P2)

**Figure 2:**
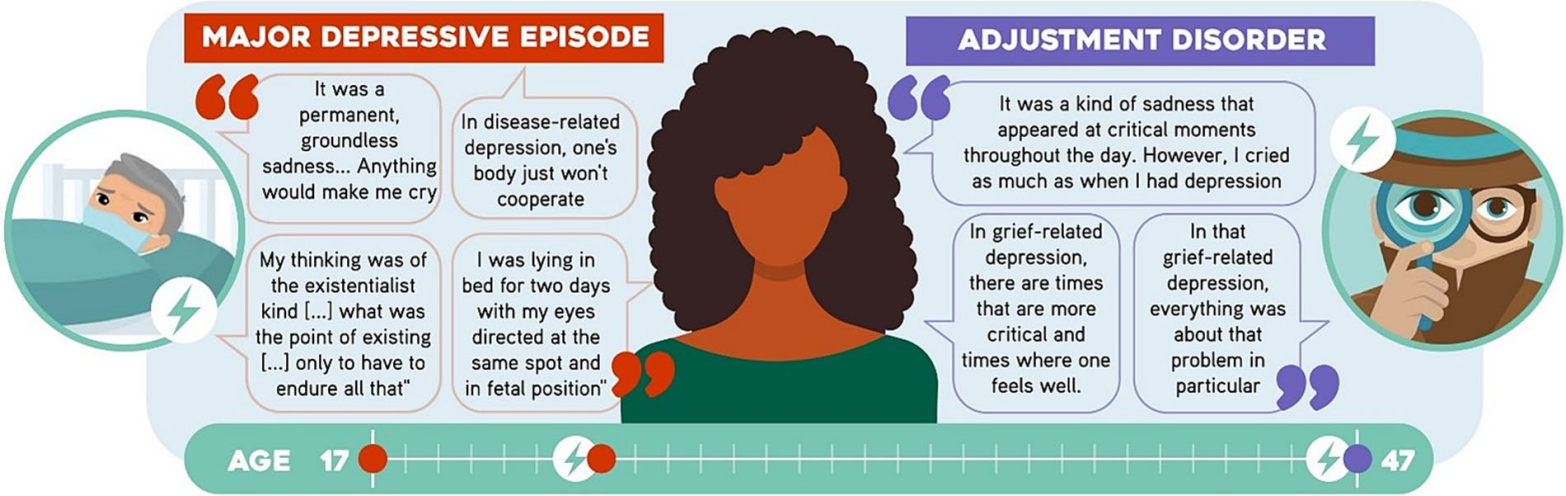
Statements by Participant 2 (P2). Some sentences said by the participant are shown.

A 49-year-old female bacteriologist with no children. She is single and holds a master’s degree and a PhD. At the age of 17, she had the first episode of what she describes as “her first time she experienced depression as a disease.” She says that it started as sadness and being prone to crying, anhedonia, social isolation, fatigue, clinophilia, existentialist ideas, hyporexia, and insomnia. She recovered completely and spontaneously after 12 months. At 29, after her father was diagnosed with cancer, she had another episode resembling the previous one but perceived as more serious: “this time I fell deeper, it felt like the disease truly kicked in this time.” During this episode, she had suicidal ideation. She then consulted a psychiatrist and was diagnosed with a *major depressive episode*. The condition was treated with fluoxetine, and continuous improvement was observed. At 47, after experiencing what she described as “harassment at the workplace,” she started to feel sad and anxious, cried frequently, had death ideation, insomnia and once asleep, frequent nightmares (Figure 2). Due to the resulting malaise and difficulty in performing her job, she consulted a different psychiatrist. *Adjustment disorder* was thus diagnosed, the treatment with fluoxetine was continued with the addition of trazodone. Her condition improved after her time off.

#### Participant 3 (P3)

**Figure 3:**
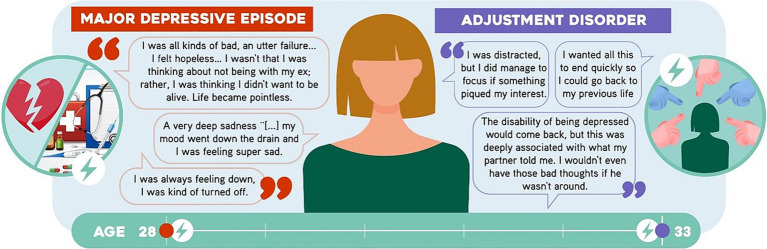
Statements by Participant 3 (P3). Some sentences said by the participant are shown.

A 36-year-old woman with no children who is a physician. She described that at 28 years of age and after breaking-up with her partner, she started to experience sadness, frequent crying, anhedonia, decreased energy, social isolation and laconicism, ideas of worthlessness, hopelessness, and death along with difficulty to concentrate and hypersomnia. This had a negative impact on her work performance, thus she consulted with a psychiatrist. She was then diagnosed with *major depressive disorder* and initiated venlafaxine treatment and psychotherapy. Her symptoms improved over the following months until they subsided and, at age 31, the antidepressant treatment was discontinued. At the age of 33, she had an internship abroad and felt she was being *bullied*. She started to feel sadness, anxiety, occasional difficulty to focus, hypobulia, ideas of worthlessness and death, insomnia, and difficulty to perform during her internship. Thus, she consulted the same psychiatrist and was diagnosed with *adjustment disorder* (Figure 3). She first started treatment with clonazepam and psychotherapy. As a result, the symptoms improved progressively during the next months.

#### Participant 4 (P4)

**Figure 4:**
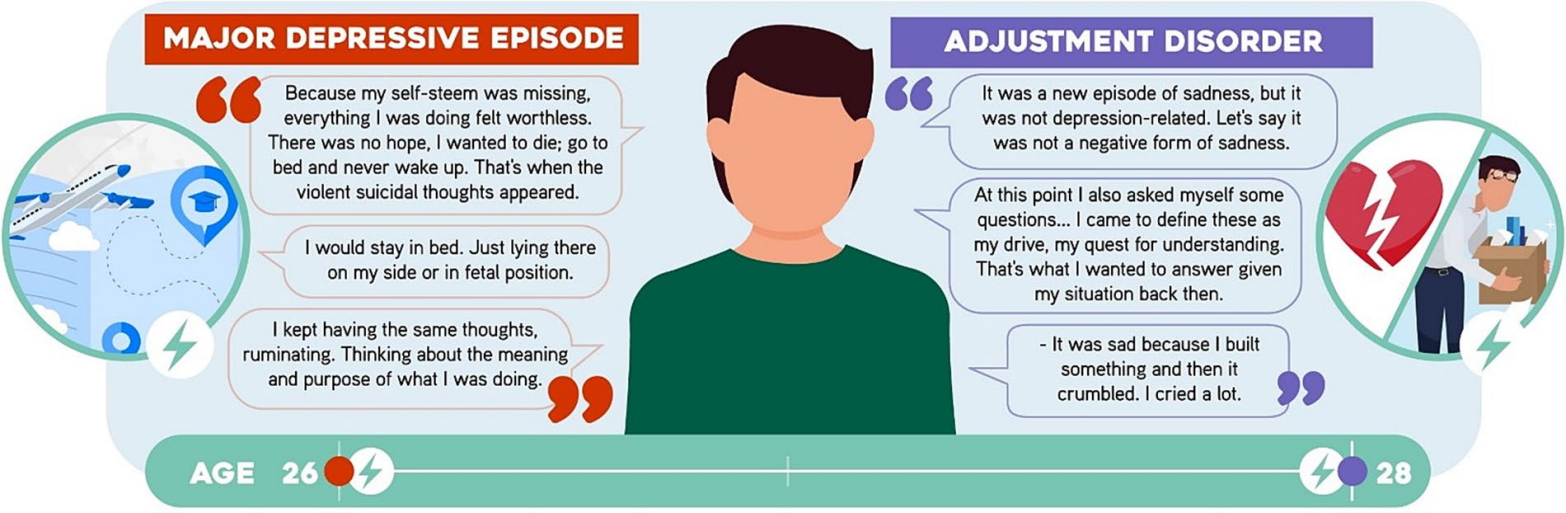
Statements by Participant 4 (P4). Some sentences said by the participant are shown.

A divorced 28-year-old male physicist with no children. He has a master’s degree and works as a university professor. During his graduate studies abroad, at the age of 26, he started to feel sadness and cry. These symptoms were accompanied by anhedonia, clinophilia, rumination, ideas of worthlessness, hopelessness, death, and suicide along with a decrease in sexual drive and insomnia. He neglected his personal hygiene and his academic performance decreased. He met a partner, whom he married. He later returned to his home country, but the symptoms persisted, thus he decided to consult a psychiatrist. He was diagnosed with a *major depressive episode*, was treated with escitalopram for 2 months and continued with psychotherapy. He had clinical recovery (“I was back to being myself at some point”). He later broke up with his partner and had difficulty to perform at work. He thus began to experience sadness, easy crying, ideas of death, decreased sexual drive, conciliation insomnia and rumination. He was assessed by his psychologist, who diagnosed an *adjustment disorder*. Psychotherapeutic treatment was thus resumed, and improvement was observed (Figure 4).

### The contrast of the two diagnoses

The four participants made it clear that the two experiences were different; none of their accounts depicted them as being similar. Having experienced MDE at first, all patients described that AD indeed felt like a different experience in seven points:

#### The attribution

Although the MDE could have been initially related to a stressful event, the participants agreed that at some point the depressive symptoms became independent of the events occurring in their daily lives. The emotion itself became a problem for them, to the point they no longer thought such emotional state was a consequence of their issues, but rather an internal problem of its own, or a disease:

*“Grieving about the loss of a partner became a secondary issue because I was already feeling very frustrated. I saw myself as a total failure: I felt I wasn’t a good daughter, girlfriend, or student. I felt like I was all kinds of bad”* (P3).

Therefore, we see that people with MDE exhibit changes in themselves and their own perception of life without focusing on the problems that they may be experiencing.

*“This sadness is different, because the other type of sadness [that of depression] is inherent, but this one is caused by external factors… When you have ‘depression disease-type’ you can leave everything behind: you do not eat or bath, you abandon your pets, your family; everything… This is when you no longer have problems, rather what you are experiencing is the problem”* (P2).

On the contrary, in AD there is an attribution towards the triggering event, which can be delimited by the onset of symptoms after the appearance of the event and the close relationship that is maintained with it throughout the course of the symptoms; participants find a clear causal relationship with the stressor:

*“The cause is important: in one case the cause is your brain’s chemistry, while in the other case the cause is an external factor that alters your biochemistry… When I was being harassed, this had an impact on my biochemistry, it did not change on its own”* (P2).

*“It is a new episode of sadness, but it’s not depression. Let us say it is not a negative form of sadness. I had this feeling that all the plans I had since my childhood shattered [and] regarding my partner, it felt as though we built something that then disappeared… so this is what caused that sadness”* (P4).

#### The theme of cognitions

The content of a person’s thoughts during MDE becomes so existential in nature that rumination regarding the meaning of life appears as well as concerns for life itself:

*“When you are depressed, you think about existence… What’s the reason for existing? What’s the point of existing? You can get stuck in that state for hours and days… so, it’s timeless and inhumane”* (P2).

*“When suffering depression, I only thought about the meaning of life. There was no other concern; I did not think about my finances or the fact that my family was having a hard time, none of that crossed my mind”* (P4).

Directing those thoughts towards all aspects of life leads to a devaluing of the subjects themselves, eventually resulting in ideas of death.

*“[When I was depressed, I had] a very strong feeling of loneliness and meaninglessness… the ideas of death came when I was lying there in my room at night, ruminating. I was continuously thinking about the kind questions you do not have an answer for. I then started to think life was pointless, and I felt like I wanted to die”* (P4).

Rumination also exists in AD, but it is directed at the triggering issue and is caused either by remembering events, thinking about solutions or not being able to see any at all. Since the focus of concern is a problem, the idea of death appears either as the solution or as a response to the emotion being triggered when thinking about it. In AD, the problem is the stressing event, not the person. In fact, those with AD do have a will to live, but without the stressor:

*“When depressed, I was often ruminating. The crucial difference lies in what it is I was ruminating about. In the case of depression, it was a very existential question for which there may be no answer. In the other case, it was all about the problems and I knew that I might come up with a solution if I spent a couple hours thinking about them”* (P4).

#### Variability in the course of symptoms

One of the features making it possible to establish a difference between MDE and AD is the course that the symptoms take on a day-to-day basis. In MDE symptoms are described as being permanent and positive emotions occur very rarely, if at all.

*“When I was depressed, I permanently felt as though I wasn’t good enough for this world. Nothing could fix that feeling, not even good happenings. It felt deeper and long-lasting”* (P3).

*“That feeling was completely permanent, I simply stayed in bed tossing and turning without doing much else in that dark room… if it were a movie, the image would not be very dynamic”* (P4).

Although sadness predominates, there comes a point where emotional life worsens and a shutting down or some sort of emotional anesthesia is felt. The four participants expressed that they reached a state which could be described as a sort of “painful athymia,” since the perception of their own lack of emotion causes suffering in itself. In other words, the possibility of having reached a state of apparent emotionlessness troubles the individual:

*“Imagine you are taken to a place in the universe where there’s nothing; it’s so empty that you feel nothing yourself. It’s indescribable, a place in the middle of nothingness, where there aren’t even colors… This was horrible for me, I tell you, I had no thoughts, but I was still aware of my presence… that really freaked me out”* (P2).

*“At first you are deeply sad: I started to feel really down, next came apathy. This felt horrible in my experience because you feel really empty, as if nothing else matters anymore, but you do not even feel bad about it. That said, it’s not like you like it either, but still, you do not feel sad about it. This feeling is too weird. In my opinion I’d be easier to be sad than apathetic because in the latter you do not feel anything anymore, neither good nor bad, and that’s really scary”* (P3).

*“I believe this is like some sort of emotional death, as if everything was only one color: black, that’s what it feels like. What’s interesting about it is that the feeling I remember from that period is that there were not many emotions”* (P4).

As for AD, there are intense symptoms of anxiety and depression, but they vary and oscillate throughout the day.

*“Symptoms fluctuate. They fluctuated based on how the pain felt. If it was severe then I was more irritable, that means it may fluctuate with pain”* (P1).

*“In contrast, in the other case that feeling was completely permanent; here it was sadness and anxiety, but more intermittent. I felt sad, very sad, but if I managed to get hooked on something or concentrate on a task, I would no longer feel sadness. It was, like, more intermittent and, I think, just as deep, but it came and went, I mean, I did feel sad, but occasionally”* (P3).

*“In this other type of sadness [AD] there is a lot of emotion, there is sadness, you do feel it. I remember that I cried a lot, and that wasn’t common when I had the other type of depression. In that one there was like some sort of emotional death”* (P4).

It is worth noting that symptoms, when present and even if they vary over time, generate the same level of suffering and dysfunction. These symptoms are like those of MDE, but the difference is that the way in which they are felt varies over time. Symptoms are similar, but their presentation differs:

*“There is sadness in both depressive crises and grief-related depression [AD], but sadness is permanent in the former. The latter does have very extreme moments of sadness as well (they feel just like those of disease-related depression), but there are also other moments when you do feel OK”* (P2).

*“In my latest episode [the patient talks about AD] I experienced very quick bouts of depression. In other words, these are the same symptoms, but they appear momentarily, as if they were flashbacks. Symptoms appear and then go away. So, there are indeed things in common with depression”* (P4).

#### Mood modulation

Symptom variability might be related to a higher or lower modulation of mood. We found that the ability to switch emotional states is compromised in those with a MDE:

*“I associate depression with deep sadness and the inability to live happy or in peace. It’s a permanent, deeply rooted state of sadness that I could not get rid of. It was always there; life was a burden for me and thus I felt I could not do anything.”* (P1).

In the case of AD, mood can be changed by strategies proposed by the individuals themselves or their surroundings. It largely depends on the presence of the stressor, since patients see it as an issue and attempt to either address it or reduce its emotional impact, all while still being able to feel pleasure.

*“I was very sad, but not depressed because I do enjoy when I go out and do other activities. Whenever possible, I would go out to dance, and I enjoyed every minute. Even when I was injured, I’d use a cane to go watch people dance and I enjoyed it… Then I changed and I was feeling down and crying, but this happened only when I had pain episodes* (P1).

*“I’d label it as depression when those feelings become the norm throughout the day. However, the latest episode [TA] had some particular sensations which would show up at very specific moments but would disappear once I switched to another activity or if I focus on something”* (P4).

#### Syndrome severity

MDE were unanimously described as utmost sadness. Participants used superlatives to represent the symptoms and express great intensity. Using that benchmark from their own experience, the participants described AD as less intense.

*“Depression is indescribable, it’s the worst. When you have disease-type depression you can leave everything behind, and in the case of grief-type depression [AD] is less intense, at times it’s hard, but at some other times you feel alright”* (P2).

For this reason, a MDE is remembered with aversion, since there is a latent fear that it will happen again. However, the accounts of AD also describe suffering and alterations in functionality, and, for this reason, professional help was sought in all cases:

*“I felt super bad again, very anxious, I could not sleep well, I could hardly concentrate, I did not want to do anything…. I wasn’t participating in class as much as before, ‘What’s wrong? Are you OK?’ people asked. So I went back to the psychiatrist, and then she diagnosed me with that adjustment disorder thing”* (P3).

#### The presence of hopelessness

One of the recurring symptoms found in the accounts of those with MDE was hopelessness. Because of the contents of one’s thoughts, the hope for living in a different state fades. In contrast, individuals with AD do hope for improvement, they may long for the moment when the stressor disappears.

*“My family and the psychologist said that the most prominent emotion they saw in me was hopelessness… I felt that I was an utter failure, that I was failing at everything and that it’d continue to be so. There was this permanent feeling that the world was too much for me… When bullying took place, I wanted it to end quickly so I could go back to normal”* (P3).

*“[In the case of MDE] hope does not exist, so you say ‘why am I going to keep dealing with these sensations? If there is no future, then why not end this once and for all since it does not make any sense…’ [In the case of AD] I also ask questions, but these are like the drive, the quest to find out how to respond to my current situation”* (P4).

#### Perception of the course

A MDE is perceived as having a more insidious course whose symptoms tend to worsen. Conversely, the course of AD is perceived as more immediate and dependent on the stressor.

*“In the case of depression, the course is slower…. [AD] goes away faster, but it also reappears faster… I went on vacation and that pressure literally started to fade and everything fell back into place. However, about 5 days before going back [to the place where I was being harassed] I started to think about it and began to feel unwell again*” (P2).

### Features of MDE and AD

The accounts of the participants show that each diagnosis has a distinct subjective experience and that its eidetic configuration may be different. We did not find any differences in terms of specific symptoms, except for hopelessness. However, we did see differences in *the mode* in which they occur. The depressive syndrome caused by MDE appears progressively and affects all aspects of an individual’s life to the point it alters the human experience. Thus, MDE can be described as an intense and progressive state of invariable and generalized shutting down of the subject’s life itself which ends up extinguishing the hope of continuing to live.

In the case of AD, the depressive (and anxious) syndrome is caused by an event, perceived as stressful, that is presented as a substrate for the individual. To this extent, AD can be outlined as a dynamic reaction to a particular situation with which a high dependency is established, which is why it courses with high variability and the hope that it will end. With that logic, the stressful event has a conative function in the subject in such a way that it forces him to mobilize, even if it is only out of desire, towards the search for a solution. In contrast, in MDE, there is little to no response to events that should direct the behavior of a human being.

These essences can influence the patient’s own understanding of the diagnosis. Participants reported how the experience of MDE is understood as a disease. This is why it is like an object appearing in the life of an individual which is even perceived as an additional burden. As for AD, although it was also introduced as a diagnosis, it is perceived as a way of naming a process that results from a given circumstance:

*“When I focus on depression, I feel like there is a lot of work to do. When I felt depression, I went [and asked myself] ‘what should I focus on? The depression or the pain?’ When I learned that it had a different label [AD] and that the pain was the reason for those symptoms I felt relieved because I could work on the emotional aspect using a different approach and because I now knew I had to focus on the pain, rather than an additional disease”* (P1).

In fact, it is possible they are expressing a stigma regarding the internal nature of MDE, and the diagnosis of AD has been perceived as reassuring because it validates the reaction to the event:

*“The diagnosis of adjustment disorder reassured me a lot. It helped me because I attribute almost everything to my disease, so it is reassuring to know that it is the result of an event, I think the name is very fitting: adjustment disorder, because we must adjust to live [with the problem]”* (P2).

## Discussion

This study found differences in the subjective experience of MDE and AD. Based on the ipsative assessment made by the participants, we found qualitative differences in the patients’ mood and emotions. Similarly, we observed that hopelessness is clinically significant for those experiencing MDE; in contrast, it is almost non-existent among those with AD. The experience of MDE was characterized by a general shutting down with no clearly discernible triggering factor. For AD, in contrast, anxiety and sadness were perceived as a direct result of a stressing event.

These findings can be approached from a phenomenological perspective since MDE is an alteration of the *structure* of existence, while AD is a particular *mode* of that existence. According to Heidegger, the basic structure of existence revolves around finding oneself in the world (*Befindlichkeit*). Upon finding oneself, one may be in a given mood (*Stimmung*) (48). Based on this notion, Fernandez suggests that depression is a loss of the Befindlichkeit or a great degradation thereof. As a result, individuals no longer see the world with the same significance with which it previously appeared to them. In other words, the core alteration experienced in depression entails a degree of severity and depth so strong that it corrodes the existence of the subject. In contrast, in AD the difference in mood and Befindlichkeit becomes apparent via a state where feelings of anxiety, sadness and self-reproach prevail and are always linked to a specific event.

It is because of this preservation of structure that emotional turmoil emerges in AD. This has been described as a form of emotionality with high variability over time. Conversely, the mood in MDE the mood is nearly static. In this regard, such variability may have a physiological correlate. Unlike MDE patients, those with AD have shown a degree of heart rate variability (HRV) which might suggest higher basal activity in the autonomous nervous system and higher ability to respond to any kind of situation (49). This is consistent with the evidence suggesting that individuals with MDE have lower reactivity to sadness and fewer moments of happiness, thus pointing to some possible insensitivity to the context, emotional detachment and failure to process rewards (50–52). In addition, their response to stressors is weakened. This has given rise to the notion of biological disconnection from life (53), which can be observed in the responses of our participants.

Our findings concern eidetic features of two human experiences and do not point to the conception of depression and AD as natural kinds. These experiences were given two different labels by an observer with a particular theoretical framework. It is thus clear that such labels, like any other clinical diagnosis, depend on the historical and cultural moment in which the naming took place (54). It is important to note that the experiences were real for the patients. Regardless of the clinical labels we may use, it is a fact that the statements obtained from the participants have an impletive sense, since there is a clear meaning in them (55). In other words, these are not just empty accounts but rather experiences filled with meaning that generate knowledge about two objects from the realm of the ideas.

Therefore, it is important to understand them as distinct concepts concerned with the achievement of two distinct goals: recovering the structure versus coping with a stressing event which alters one’s mode. Therefore, a specific label is important, since the consequences of having a name could have a crucial impact: emphasizing antidepressant treatment would not be the same as empowering a person to find solutions and coping mechanisms. Indeed, in our study, receiving a diagnosis of AD had a positive effect on how the subjects perceived their own experience. The absence of a disorder or “disease” (as they claimed) was seen as a relief and highlighted the need to focus their efforts towards developing a mechanism for coping with the stressing event. Probably having been diagnosed with AD and MDE may have influenced the memory of what was experienced and we could unveil the phenomenon recognized as the “looping effect” of human classes, described by Ian Hacking (56); insofar as the designation of a category, in this case, of the psychiatric classification (a socio-linguistic practice in the encounter with the clinician) could determine the behavior of the patients. Studying the full impact of diagnoses may have on patients’ lives is beyond the scope of this article and could be investigated in the future.

Distinguishing AD from MDE is a valuable clinical exercise, since overdiagnosing depression has become common during recent years and has led to antidepressants overprescription and the reduction of psychotherapy remissions (57, 58). If the reaction to an event is at the core of AD, focusing on antidepressants will not be necessary since there is no empirical evidence supporting their effectiveness (59). In contrast, using psychotherapy to modulate the interaction with the stressor would be crucial (60). This reasoning about coping with the problem is what some patients perceive as insufficient when they undergo treatment with antidepressants (61) which could hinder the understanding of the episode as a structural change requiring psychiatric drugs.

It is worth noting that the label of AD also allows clinicians to follow-up on the patients and does not imply the absence of psychopathology, since we did see significant malaise among the patients. It is necessary to offer specific treatment and actively monitor the evolution of symptoms. It has been suggested that people lacking appropriate autonomic reactivity are more likely to have MDE (62), which shows the importance of measuring this reactivity. Even in the face of extreme events such as the loss of a loved one, the insensitivity of the patients towards the context may suggest the existence of distinct kinds of patients with a worse prognosis given the amount and duration of depressive symptoms (63). For this reason, there is likely a transition from AD to MDE when the stressor ceases to be a substrate and the emotional phenomena become independent. A clinical transition into a state of psychopathology is possible upon losing that attribution along with the high reactivity. In fact, our patients stated that the existence of this apparent transition is what prompted them to consult. This variation over time at specific dimensions of symptom patterns is a fertile field of research where multiple modeling techniques can be used to detect critical transitions (64) which could include loss of reactivity and attribution as an alarm signal.

We believe that returning to the experiences of the patients is a strength of this study. The accounts provided by the patients and the distinction resulting from their self-observation contributes to the description of symptoms in psychiatry. Ultimately, experiences are the input allowing clinicians to make decisions during their practice. Also, the phenomenological description of AD has been based on coping with stressors (65), rather than on the clinical manifestations that allow its differentiation from other disorders and therefore this manuscript responds to the call to deepen the psychopathology of this disorder (66, 67), which can enrich and refine the diagnostic criteria in the future so that the label of AD can be better grounded. We propose AD as a commutative possibility of the variations of the depressive symptoms. Nevertheless, it is necessary to mention certain limitations. The main one is the small sample size. Arguing that this size is sufficient for such a complex and boundless might lead to error, but in qualitative research there is no standard sample size. In phenomenological and narrative studies, recommended sample size can vary from 3 to 25 participants (68), and even one person may also be informative (40). The balance between the number of sources of information and the comprehension of that information, although difficult to achieve, is influenced by the topic being studied and the diversity being captured. We believe that the depth of our understanding was adequate to obtain the description of the phenomena in these patients, which had a feature that was difficult to find and that was the formal diagnosis of AD and MDE. We were able conduct an in-depth dialogue that would allow us to follow an idiographic and ipsative logic with them to we reach theoretical saturation. This implies also that patients with another level of abstraction and education or with milder forms of the disorder that did not have as much clinical detail may not be represented in our findings. Another limitation has to do with the researchers; this phenomenological study was conducted by clinicians with general training in qualitative and phenomenological research which could limit the depth of the analysis if it were taken to a pure philosophical field. It is also possible that there is a recall bias due to the ability of the participants to narrate their experience; although it is irreparable because it was precisely the intention to understand the episodes from the first-person perspective, the exploration of other sources of information such as family members and clinicians could have contributed to a greater precision of the statements; exploration of these other perspectives could lead to new research that complements our findings.

## Conclusion

The ipsative assessment of the patients with MDE and AD showed differences which suggest that these conditions are experienced in substantially different manners. Thus, the harsh and progressive state of general shutdown of the subject’s own life with little response to events could be labeled as MDE. AD, in turn, would be the dynamic reaction attributed to a stressing event. In this scenario the course of the symptoms is highly variable given their dependency on that event. In addition, there is the hope that the event will end.

## Data availability statement

The original contributions presented in the study are included in the article. Further inquiries can be directed to the corresponding author.

## Ethics statement

The studies involving humans were approved by Ethical Review Board of the School of Medicine of the University of Antioquia (Approval Act 008 of May 21, 2020). This Committee was constituted by Resolution of the Universidad de Antioquia School of Medicine at its meeting of May 30, 2008, minute 177. The Committee is identified to the National Institutes of Health (NH) with the codes IORG0010323 and IRB00012257. The Committee’s Federal Assurance Code is: FWA00028264. The studies were conducted in accordance with the local legislation and institutional requirements. The participants provided their written informed consent to participate in this study. Written informed consent was obtained from the individual(s) for the publication of any potentially identifiable images or data included in this article.

## Author contributions

JZ-O: Conceptualization, Formal analysis, Funding acquisition, Investigation, Methodology, Visualization, Writing – original draft. MJ-B: Formal analysis, Investigation, Methodology, Writing – review & editing. MF: Formal analysis, Supervision, Writing – review & editing.
