# Supplementary material for: “I was very sad, but not depressed”: phenomenological differences between adjustment disorder and a major depressive episode
Source: Front Psychiatry. 2023 Dec 11;14:1291659. doi: 10.3389/fpsyt.2023.1291659 (PMC10749326; doi:10.3389/fpsyt.2023.1291659)
Supplement: Supplementary file 1 [file Table_1.pdf]

COREQ (COnsolidated criteria for REporting Qualitative research) Checklist

(

Topic	Item No.	Guide Questions/Description	Reported on Page No.
**Domain 1: Research team and reflexivity**			
Personal characteristics			
Interviewer/facilitator	1	Which author/s conducted the interview or focus group?	✓
Credentials	2	What were the researcher's credentials? E.g. PhD, MD	✓
Occupation	3	What was their occupation at the time of the study?	✓
Gender	4	Was the researcher male or female?	✓
Experience and training	5	What experience or training did the researcher have?	✓
Relationship with participants			
Relationship established	6	Was a relationship established prior to study commencement?	✓
Participant knowledge of the interviewer	7	What did the participants know about the researcher? e.g. personal goals, reasons for doing the research	✓
Interviewer characteristics	8	What characteristics were reported about the inter viewer/facilitator? e.g. Bias, assumptions, reasons and interests in the research topic	✓
**Domain 2: Study design**			
Theoretical framework			
Methodological orientation and Theory	9	What methodological orientation was stated to underpin the study? e.g. grounded theory, discourse analysis, ethnography, phenomenology, content analysis	✓
Participant selection			
Sampling	10	How were participants selected? e.g. purposive, convenience, consecutive, snowball	✓
Method of approach	11	How were participants approached? e.g. face-to-face, telephone, mail, email	✓
Sample size	12	How many participants were in the study?	✓
Non-participation	13	How many people refused to participate or dropped out? Reasons?	✓
Setting			
Setting of data collection	14	Where was the data collected? e.g. home, clinic, workplace	✓
Presence of non-participants	15	Was anyone else present besides the participants and researchers?	✓
Description of sample	16	What are the important characteristics of the sample? e.g. demographic data, date	✓
Data collection			
Interview guide	17	Were questions, prompts, guides provided by the authors? Was it pilot tested?	✓
Repeat interviews	18	Were repeat inter views carried out? If yes, how many?	✓
Audio/visual recording	19	Did the research use audio or visual recording to collect the data?	✓
Field notes	20	Were field notes made during and/or after the inter view or focus group?	✓
Duration	21	What was the duration of the inter views or focus group?	✓
Data saturation	22	Was data saturation discussed?	✓
Transcripts returned	23	Were transcripts returned to participants for comment and/or	✓

Topic	Item No.	Guide Questions/Description	Reported on Page No.
		correction?	
**Domain 3: analysis and findings**			
Data analysis			
Number of data coders	24	How many data coders coded the data?	✓
Description of the coding tree	25	Did authors provide a description of the coding tree?	✓
Derivation of themes	26	Were themes identified in advance or derived from the data?	✓
Software	27	What software, if applicable, was used to manage the data?	✓
Participant checking	28	Did participants provide feedback on the findings?	✓
Reporting			
Quotations presented	29	Were participant quotations presented to illustrate the themes/findings? Was each quotation identified? e.g. participant number	✓
Data and findings consistent	30	Was there consistency between the data presented and the findings?	✓
Clarity of major themes	31	Were major themes clearly presented in the findings?	✓
Clarity of minor themes	32	Is there a description of diverse cases or discussion of minor themes?	✓

Developed from: Tong A, Sainsbury P, Craig J. Consolidated criteria for reporting qualitative research (COREQ): a 32-item checklist for interviews and focus groups. *International Journal for Quality in Health Care*. 2007. Volume 19, Number 6: pp. 349 – 357
